# Excitotoxic neurodegeneration is associated with a focal decrease in metabotropic glutamate receptor type 5 availability: an *in vivo* PET imaging study

**DOI:** 10.1038/s41598-019-49356-x

**Published:** 2019-09-09

**Authors:** Melissa Crabbé, Nina Dirkx, Cindy Casteels, Koen Van Laere

**Affiliations:** 1 Nuclear Medicine and Molecular Imaging, Department of Imaging and Pathology, KU Leuven and University Hospitals Leuven, Leuven, Belgium; 2 MoSAIC - Molecular Small Animal Imaging Centre, KU Leuven, Leuven, Belgium

**Keywords:** Diseases of the nervous system, Experimental models of disease

## Abstract

Metabotropic glutamate receptors (mGluRs) have been proposed as promising therapeutic targets to correct the dysregulated glutamate signaling, associated with neurodegenerative pathologies. Of all mGluR subtypes, especially mGluR5 acts as a modulator of glutamate-induced excitotoxicity. To study the behavior of mGluR5 following localized excitotoxicity, we utilised a pharmacological model that portrays exacerbated neuronal glutamate release, mediated by the endogenous excitotoxin quinolinic acid (QA). Using longitudinal positron emission tomography (PET) with [^18^F]FPEB, we investigated cerebral changes in mGluR5 following striatal QA-lesioning. Behavioral tests were executed to monitor motor and cognitive performance. Decreased mGluR5 binding potential (*BP*_*ND*_) was found in the affected striatum and globus pallidus of QA-lesioned rats at week 3, and further decreased at week 7, as compared to sham-injected controls. mGluR5 availability in the ipsilateral nucleus accumbens was significantly decreased at 7 weeks post-injection. QA rats performed significantly worse on motor coordination and balance compared to control rats. Correlation analysis indicated a positive correlation between striatal mGluR5 *BP*_*ND*_ and rotarod performance whereas print width of the unaffected forepaws showed a positive relation with mGluR5 *BP*_*ND*_ in the contralateral motor cortex. Together, our results suggest decreased mGluR5 availability to be related to excitotoxin-induced neurodegeneration and symptomatology although late stage effects do indicate possible cortical mGluR5-mediated effects on motor behavior.

## Introduction

Glutamate is the dominant excitatory neurotransmitter in the brain and plays a crucial role in the central nervous system (CNS) by acting through two types of receptors, the ionotropic (iGluR) and metabotropic (mGluR) glutamate receptors. Glutamatergic dysfunction, leading to excitotoxic neuronal death, is a common neuropathological pathway in several CNS disorders, including Parkinson’s (PD) and Huntington’s disease (HD)^1,2^. Chronic glutamate-mediated neurotoxicity through overstimulation of iGluRs (such as the N-methyl-D-aspartate (NMDA) receptor), but also mGluRs, has been implicated in the disease onset and progression of several neurodegenerative diseases^3–5^. To date, the mechanisms that underlie the disease progression of these neurodegenerative diseases remain unclear, complicating the quest for a disease-modifying drug, which would be able to cure or slow down the disorder.

Out of the 8 types of mGluRs, mGluR5 is a transmembrane G-protein-coupled receptor linked to phospholipase C and inositol 1,4,5-triphosphate and increases intracellular calcium levels following its activation^6^. mGluR5 has been targeted for potential treatment of various neurodegenerative disorders and possesses some favorable characteristics. First, mGluR5 is involved in the regulation of normal locomotor activity. Hence the receptor may be centrally involved in neurodegenerative movement disorders^7,8^. In a physiological state, glutamatergic signaling is a key factor in the normal functioning of the basal ganglia – thalamus – cortical loop, thereby mediating voluntary motor function^9^. mGluR5 modulation was shown to improve motor function in several movement disorders both clinically and in rodent models^10,11^. Second, mGluR5 may act as a co-receptor to mutant huntingtin, amyloid-β plaques and α-synuclein oligomers, providing a direct link between mGluR5 and proteinopathies^12–14^. mGluR5 activation may facilitate the activation of NMDA receptors through the Homer/PSD95/Shank protein complex in dendritic spines, and this way play a permissive role in neuronal excitotoxicity^15^. Accordingly, mGluR5 positive allosteric modulators (PAMs) were shown to be predominantly neurotoxic whereas negative allosteric modulators (NAMs) have shown neuroprotective properties against excitotoxic degeneration, although clinical trials have shown no major benefit so far^16–19^. Third, the topographical organization of neuronal mGluR5 expression is restricted to cerebral regions affected by neurodegenerative diseases, such as the striatum, cerebral cortex and hippocampus^20^.

The striatum is one of the primarily affected structures in several movement disorders and is known to play a cardinal role in the pathophysiology of both motor and non-motor symptoms. To date, however, the effect of excitotoxicity and subsequent post-synaptic dysfunction of striatal medium spiny neurons (MSN) on mGluR5 availability has not been researched yet. Localized dysfunction and degeneration of MSNs has been modeled by neurotoxin models such as quinolinic acid (QA), a model for excitotoxic lesioning and HD. We therefore investigated the effect of QA lesioning on regional mGluR5 levels *in vivo*, employing 3-[^18^F]-fluoro-5-(2-pyridinylethynyl) benzonitrile ([^18^F]FPEB) microPET in relation to behavioral measures and immunohistochemistry.

## Materials and Methods

### Animal model

All animal experiments were performed in accordance with the European Communities Council Directive 2010/63/EU and approved by the local Animal Ethics Committee of the KU Leuven (P147/2014). *In vivo* experiments were conducted on 15 male Sprague-Dawley rats (on average 10 weeks old; body weight range at the start of the experiment 320.6 ± 13.1 g). Animals had free access to pellet food and tap water, and were under a 12 h light/dark cycle. Stereotactic quinolinic acid (QA) injections into the left striatum were executed in accordance to a protocol described previously^21^. In short, animals were injected with 2 µl containing either QA (n = 10; 120 nmol dissolved in 0.9% saline solution) or saline solution (control group, n = 5), using following coordinates for the striatum: anteroposterior (AP) +0.2, lateral (LAT) +2.8, dorsoventral (DV) −4.5. QA- and saline-injected rats will be mentioned hereafter as QA and control groups, respectively. For histology, an additional cohort of male Sprague-Dawley rats were included of the same age with a body weight range 296.1 ± 16.5 g.

### Small-animal PET imaging

mGluR5 imaging was performed using [^18^F]FPEB (3-[^18^F]-fluoro-5-(2-pyridinylethynyl)benzonitrile)^22–24^. [^18^F]FPEB was synthetized on-site using the nitro-precursor obtained from ABX (Advanced Biochemical Compounds, Radeberg, Germany), as previously described^25^. PET experiments were performed on a lutetium oxyorthosilicate detector-based small-animal tomograph (FOCUS-220; Siemens/Concorde Microsystems, Knoxville, TN, USA). This system has a 1.35 mm full-width at half-maximum (FWHM) transaxial resolution. Data were collected in a 128 × 128 × 95 matrix with a pixel width of 0.475 mm and 0.795 mm slice thickness.

Before and during PET imaging, rodents were anesthetized using 2.5% isoflurane in 100% oxygen (1.5 l/min flow rate) and temperature was maintained at ±37 °C. Tail veins were catheterized for injection of 18.2 ± 2.2 MBq [^18^F]FPEB. Dynamic 60-min scans were initiated simultaneously with [^18^F]FPEB injection. Scans were conducted at three time points – in the last time point (7 weeks), there was a technical issue with the scans of three QA rats and were not included in the PET data analysis at this time point.

### PET image reconstruction and data processing

List-mode data were reconstructed in 21 frames (4 × 15, 4 × 60, 5 × 180 and 8 × 300 seconds) using an iterative maximum a posterior probability (MAP) algorithm with ordered subsets (18 iterations, 9 subsets; fixed resolution: 1.5 mm) and attenuation corrected by means of a ^57^Co-transmission scan, conducted prior to the dynamic scan.

PET images were normalized to an in-house rat brain template in Paxinos stereotactic space^26^. Parametric non-displaceable binding potential (*BP*_*ND*_) images of [^18^F]FPEB were generated by using Ichise’s original multi-linear reference tissue model (MRTM0), with the cerebellum as reference tissue^27^.

Voxel-wise analysis was performed using Statistical Parametric Mapping 12 (SPM12, Wellcome Department of Cognitive Neurology, London, United Kingdom). Spatially normalized images were masked to exclude extra-cerebral signal and smoothed with an isotropic Gaussian kernel of 1.6 mm. SPM analysis was performed using a 0.8 relative threshold of mean image intensity. T-maps were interrogated at a p_height_ ≤ 0.005 (uncorrected) peak level and extend threshold of k_E_ > 200 voxels (1.6 mm^3^). A multifactor design including ‘condition’ (QA vs. control) × ‘time’ (baseline, 3- and 7-weeks) was used for voxel-wise analysis.

VOI-based analysis was executed using a predefined VOI map including the bilateral striatum, nucleus accumbens, hippocampus and cortex (PMOD, version 3.4, PMOD Technologies LTD, Zurich, Switzerland). In addition, we performed a voxel- and VOI-based correlation analysis between (1) mGluR5 binding, (2) lesion volume, and (3) behavioral outcomes.

### Behavioral testing

All behavioral tests were executed during the light phase of the 12 h light/dark cycle at baseline, 3 and 7 weeks post-injection. Tests were performed on two consecutive days prior to PET imaging, in the same order, and on similar time points throughout the day. All rats were evaluated for motor dysfunction with the rotarod^28^ and CatWalk test^29^ while cognitive performance was evaluated using the Novel Object Recognition Test^30^.

#### Rotarod

Prior to lesioning, rats were trained for 5 consecutive days on an accelerating rotarod (4 to 40 rounds per minute (RPM) in 5 minutes). Every day, each rat was trained 3 times, with a minimum of 20 minutes between each run. Rodents were considered trained if they could remain for >90 sec on the accelerating rod, after which baseline was taken. After surgery, rats were subjected to the same accelerating protocol and latency to fall off (in seconds) was recorded.

#### Catwalk

The Catwalk™ (Noldus Information Technology, Wageningen, The Netherlands) has been proven useful to detect gait disturbances in animal models of neurodegenerative diseases and allows for semi-automated quantification of a number of locomotor features, such as stride length and interlimb coordination. Animal training and performance of the catwalk test was executed as described previously^25^.

#### Novel object recognition Test (NORT)

To investigate cognitive impairment involving memory and spatial navigation, we performed the NORT, following a previously described protocol^25^. The recognition index (RI) was calculated by (T_B/C_/(T_A_ + T_B/C_))x100, with T_A_ as the time spent exploring the familiar object A and T_B/C_ as time spent exploring novel object B (short-term memory) or C (long-term memory).

### Histology and stereological quantification

An additional cohort of 8 rats (QA: n = 4; saline: n = 4) was included for immunohistochemistry and sacrificed at 3 weeks post-injection.

Rats were sacrificed using a sodium pentobarbital overdose (60 mg/kg, *i.p*., Nembutal®, Ceva Santé Animale, Brussels, Belgium) after which intracardial perfusion was performed with 10% glucose in phosphate-buffered saline (PBS), followed by 4% paraformaldehyde in PBS. After 24 h post-fixation, samples were kept at 4 °C until further processing. Sectioning and subsequent immunostaining were performed in accordance with Van der Perren *et al*.^28^. In short, antibodies targeting either neuron-specific protein (NeuN; rabbit polyclonal 1:1000, ABN78, Millipore, Massachusetts, USA) or mGluR5 (1:1000, AB5675, Millipore), and biotinylated anti-rabbit IgG as a secondary antibody (1:300, DakoCytomation, Belgium) were used, followed by incubation with streptavidin–horseradish peroxidase complex (1:1000, DakoCytomation), and employing Vector SG (SK-4700, Vector Laboratories, CA, USA) as a chromogen. The stereological volume of NeuN-immunoreactive neurons in the striatum was determined through the Cavalieri method as described previously^28^.

For fluorescent double staining, sections were rinsed three times in PBS and then incubated overnight in PBS with 0.1% triton X-100 and 10% donkey serum. The following antibodies were used: goat anti-iba-1 (polyclonal 1:500, ab107159, Abcam, UK), mouse anti-NeuN (1:500, MAB377, Millipore), and rabbit anti-mGluR5 (1:500, AB5675, Millipore). Following 3 rinses in PBS with 0.1% triton X-100, sections were incubated for 2 h in the dark with fluorochrome-conjugated secondary antibodies: donkey anti-goat Alexa 594 (1:500, A-21432, Molecular ProbesTM, Invitrogen, Belgium), donkey anti-mouse Alexa 568 (1:500, A-31571, Invitrogen), donkey anti-rabbit Alexa 488 (1:500, A-21206, Invitrogen), followed by counterstaining with DAPI and mounting with mowiol. The staining was visualized using the Axio Scan.Z1 slides scanner (Zeiss, Belgium). Image contrast was adjusted using the Fiji processing package to improve image quality^31^.

### General statistics

Reported values are described as the mean ± standard deviation. Conventional statistics of VOI and behavioral data were executed using 2-way repeated-measures analysis of variance (ANOVA) and non-parametric Mann-Whitney U tests (PRISM 7, GraphPad Software, Inc., CA, USA). Tukey post-hoc tests were utilized to correct for multiple comparisons. A correlation analysis was performed using Spearman rank correlation on significantly altered findings from [^18^F]FPEB PET, behavioral tests and histological analysis. *P*-values < 0.05 were accepted as statistically significant.

## Results

### Small-animal [^18^F]FPEB PET imaging

**Figure 1:**
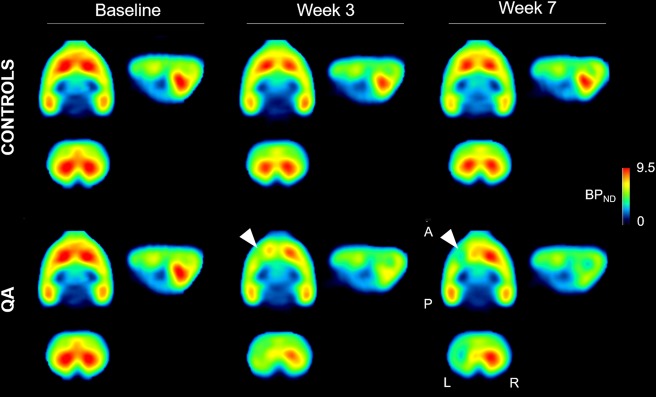
Average orthogonal [^18^F]FPEB *BP*_*ND*_ in saline-injected controls rat (n = 5) and QA rats (n = 10). A distinct decrease in [^18^F]FPEB *BP*_*ND*_ is notable following lesioning of the striatum (left hemisphere, white arrows). The intersection point has been set to the mid-striatal level in the lesioned hemisphere. Color bars indicate binding potential (*BP*_*ND*_) values for the radioligand. Abbreviations: A, anterior; P, posterior; L, left; R, right; QA, quinolinic acid; *BP*_*ND*_, non-displaceable binding potential.

Mean cross-sectional PET images of [^18^F]FPEB binding in the rat brain over time are shown in Fig. 1. Regional [^18^F]FPEB uptake is high in the striatum, hippocampus, and cortex, in accordance with the known brain distribution of mGluR5^20,27^.

**Figure 2:**
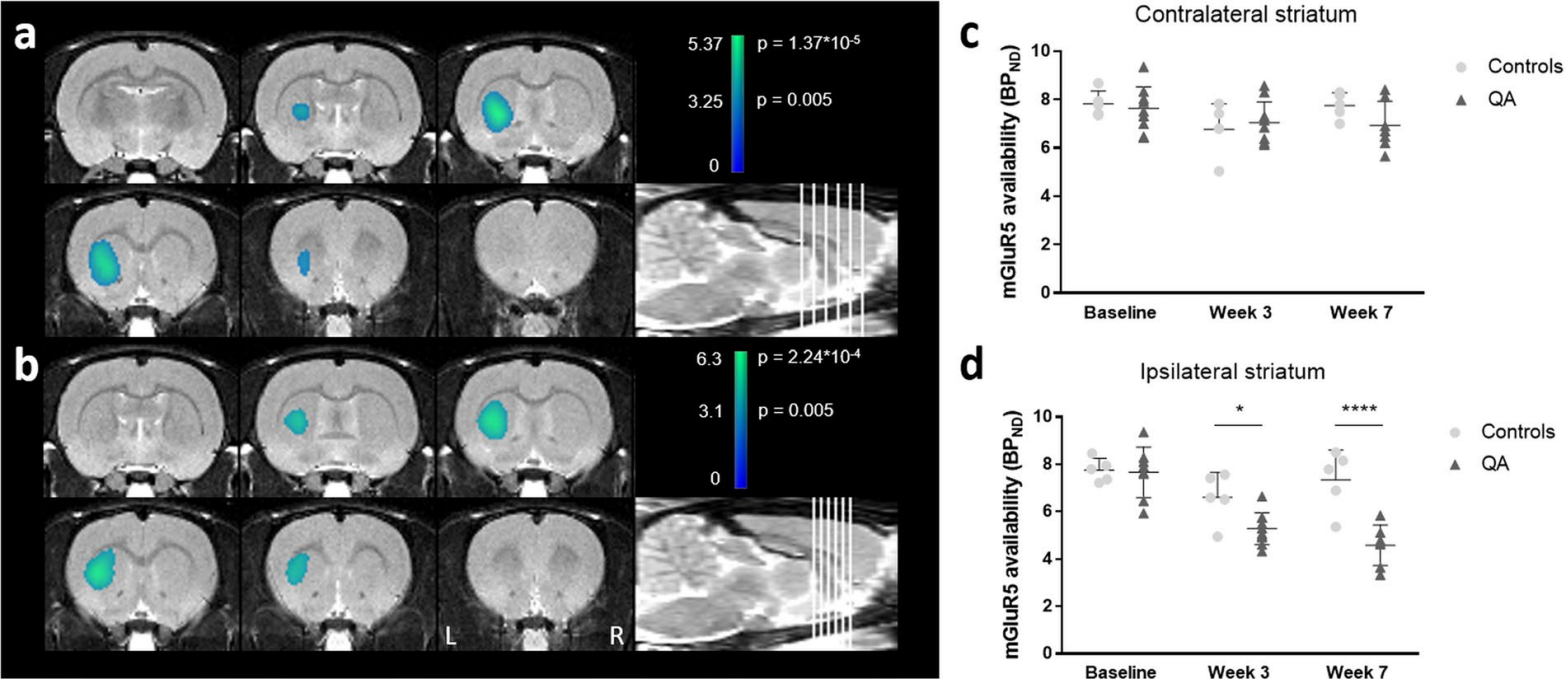
Longitudinal comparison of [^18^F]FPEB uptake in QA-lesioned rats and their respective controls. **(a**,**b)** Coronal brain sections indicate overlays on the regions with significantly decreased [^18^F]FPEB binding potential (*BP*_*ND*_) at respectively 3 and 7 weeks post-lesioning. Significant clusters are shown using a T-statistic color scale, which corresponds to the level of significance at the voxel level. Images are in neurological convention. L, left; R, right. **(c**,**d)** Scatter plots showing *BP*_*ND*_ values for the mGlu5 receptor, determined by VOI analysis. Note that n = 7 for QA rats at the 7-week time point as 3 rats were not included in the PET analysis. All data are shown as mean ± SD. 2-way ANOVA, *p < 0.05, ****p < 0.0001. Abbreviations: QA, quinolinic acid.

VOI-based analysis indicated decreased mGluR5 availability in the affected striatum of QA rats over time, both at the 3- and 7-week time point (W3: −21.5 ± 7.6%, *p* = 0.02; W7: −40.0 ± 7.2%, *p* < 0.0001) in comparison with controls. In contrast, tracer uptake was not significantly different in the non-lesioned striatum. Moreover, reduced binding was shown in the ipsilateral nucleus accumbens at week 7, as compared to control animals (W3: −11.8 ± 9.6%; W7: −25.7 ± 10.0%; *p* = 0.50 and *p* = 0.04, respectively). VOI-based *BP*_*ND*_ values in both the lesioned and non-lesioned striatum are given in Table 1. The voxel-based SPM analysis confirmed the previous VOI findings, showing significantly reduced mGluR5 *BP*_*ND*_ values in a cluster comprising the ipsilateral striatum and globus pallidus at week 3 and 7 (mean decrease at Paxinos coordinate peak maximum: −31.3 ± 11.0%; *p*_uncorr_ = 2.24 * 10^−4^ at week 3 and −38.3 ± 13.3%; *p*_uncorr_ = 1.37 * 10^−5^ at week 7; Fig. 2). Detailed cluster peak locations and *p*-values of the voxel-based categorical analysis using SPM are shown in Table 2.

**Table 1:** [^18^F]FPEB binding potential in the quinolinic acid-lesioned striatum as measured by *in vivo* microPET.

	Ipsilateral striatum		Contralateral striatum	
	Controls	QA	Controls	QA
Baseline	7.76 ± 0.50	7.66 ± 1.07	7.84 ± 0.53	7.68 ± 0.97
Week 3	6.61 ± 1.05	5.08 ± 0.80^*^	6.77 ± 1.07	7.05 ± 0.84
Week 7	7.34 ± 1.16	4.58 ± 0.86^◊^	7.24 ± 1.33	6.50 ± 1.48

Non-displaceable binding potential (*BP*_*ND*_) of the radiotracer are represented in both affected (ipsilateral)- and non-affected contralateral striata at the three time points studied. Data are reported as mean ± SD. *Significantly different from the corresponding hemisphere in the controls rats. *p < 0.05, ^◊^*p* < 0.0001; 2-way repeated-measures ANOVA with Tukey correction for multiple testing.

**Table 2:** Overview of SPM findings.

Peak location for the cluster in the group comparison and correlation analysis (at p_height_ < 0.005 uncorrected, K_E_ > 200)									Name
	Cluster-level		Voxel-level		Intensity difference (%)	Coordinates			
	p_corr_	K_E_	T	p_uncorr_		x (LAT)	y (AP)	z (DV)	
**Categorical analysis: [** ^**18**^ **F]FPEB**									
QA_BL-3w_ < Saline_BL-3w_	0.11	2173	5.4	2.2 * 10^−4^	−31.3 ± 11.0%	3.2	0.2	−6.2	Ipsilateral striatum and lateral globus pallidus
QA_BL-7w_ < Saline_BL-7w_	0.02	1209	6.3	1.4 * 10^−5^	−38.3 ± 13.3%	3.8	0.2	−5.4	Ipsilateral striatum and lateral globus pallidus
**Correlation analysis**									
Positive correlation with latency to fall on rotarod	0.002	5471	6.9	2.0 * 10^−5^		5.0	1.8	−4.2	Ipsilateral striatum and primary somatosensory cortex
						3.0	1.2	−3.2	
						4.4	2.6	−4.0	
Positive correlation with print width of unaffected paw on Catwalk	1.0 * 10^−4^	2326	14.0	4.1 * 10^−6^		−4.6	−1.6	−2.6	Contralateral primary motor and somatosensory cortex
						−4.2	0.2	−2.4	
						−1.8	−1.2	−1.4	

*P*_corr_ at the cluster level = chance (*p*) of finding a cluster with this or greater size (K_E_), corrected for search volume; K_E_ = cluster extent; T = measures of the statistical significance; *P*_uncorr_ at voxel level: the chance (*p*) of finding a voxel with this or a greater height (T-statistic), uncorrected for search volume; Intensity difference = % difference at the voxel level of QA rats in comparison to controls; X = lateral distance in mm from the midline (negative values on the right side); Y = anteroposterior location relative to Bregma (negative values: posterior to Bregma); Z = dorsoventral position (based on the Paxinos stereotactic atlas).

### Behavioral outcome

**Figure 3:**
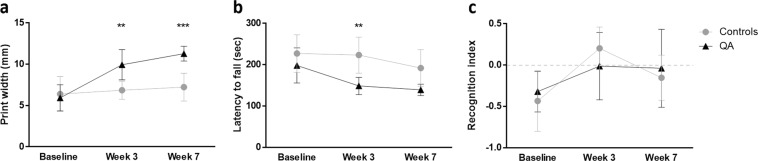
Behavioral outcomes in lesioned rats (n = 10) and their respective controls (n = 5). (**a**,**b)** Effect of QA-lesioning on motor performance as measured by the Catwalk gait analysis (**a**) and rotarod performance (**b**). Both a and b show that QA-lesioning resulted in significant motor deficiencies. **(c**) Novel object recognition test. QA rats did not depict significantly altered memory function. **p < 0.01, ***p < 0.001; 2-way ANOVA. Data are indicated as mean ± SD. Abbreviations: QA, quinolinic acid.

QA-lesioning resulted in altered motor function, as demonstrated by detailed gait analysis utilizing the CatWalk test. A general increase in print width of the ipsilateral, unaffected forepaw was noted in QA rats post-lesioning, in an attempt by the animals to stabilize gait (% increase print width: +44.8 ± 18.2% and +56.0 ± 8.9%, respectively; *p* < 0.01 and *p* < 0.001, respectively; Fig. 3a).

Lesioning of the striatum resulted in a decreased latency to fall in the QA rats, suggesting reduced endurance and/or motor-planning. Significance was reached only at the 3-week evaluation when comparing to controls (latency to fall: 148.5 ± 20.9 vs. 223.3 ± 43.6 sec; *p* < 0.01; Fig. 3b). Additionally, rotarod performance worsened significantly for QA rats over time (baseline: 198.1 ± 42.4 sec vs. week 7: 139.1 ± 13.6 sec; *p* = 0.02).

The novel object recognition test did not indicate any differences in short- or long-term memory between QA-lesioned and sham-lesioned animals (Fig. 3c).

### [^18^F]FPEB correlation analysis

**Figure 4:**
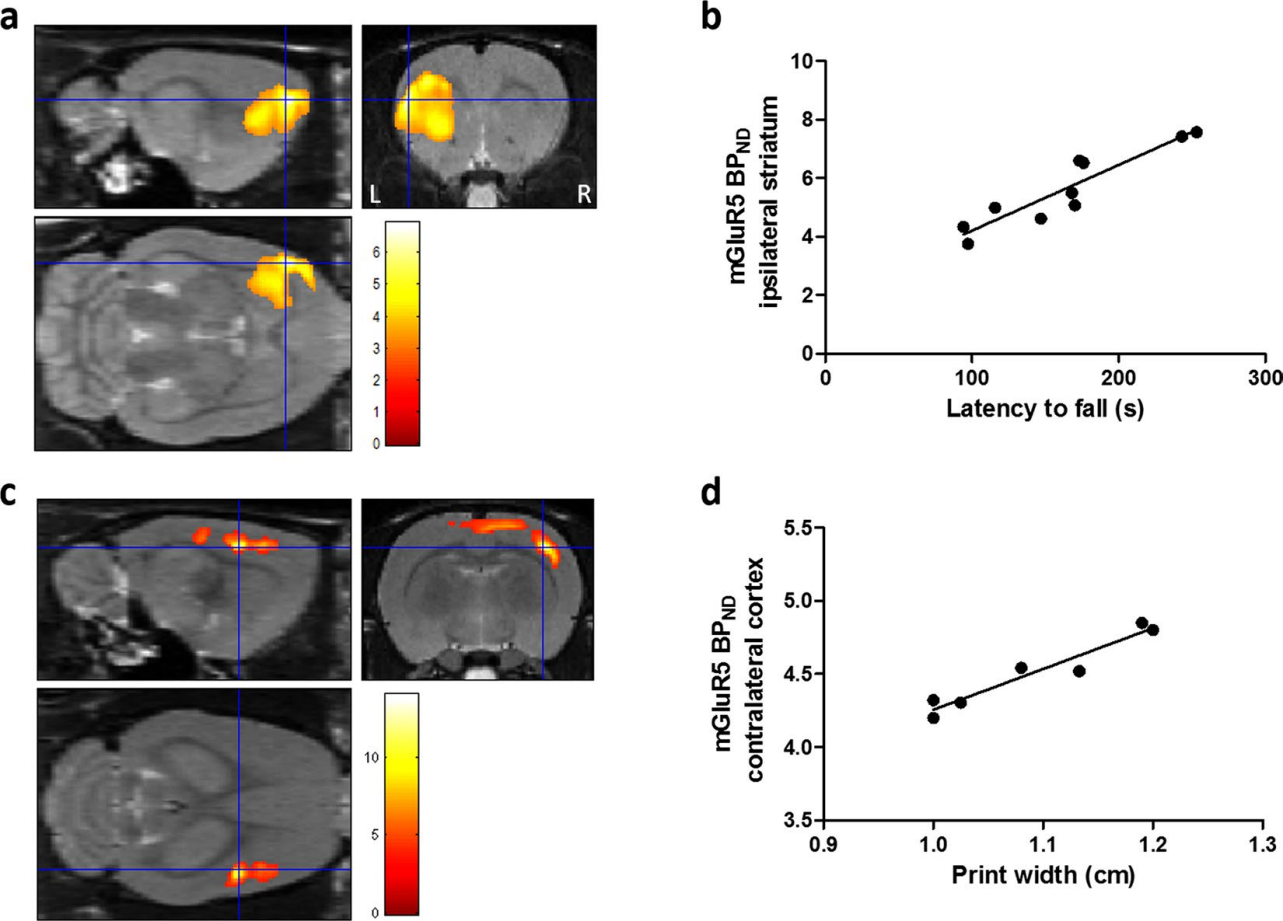
Voxel-based correlation analysis. (**a**,**b)** A positive correlation was shown between latency to fall (rotarod) and mGluR5 binding potential (BP_ND_) values at 3 weeks post-lesioning with the QA rat population. **(c**,**d)** Print width (Catwalk) correlated positively to mGluR5 *BP*_*ND*_ values at 7 weeks post-lesioning. Left panel: Statistical parametric maps showing an overlay of the clusters with a significant correlation in QA rats. Significance is shown with a T-statistic color scale, which corresponds to the level of significance at the voxel level. Right panel: Scatter plots of voxel-based correlation analysis in QA rats, indicating mGluR5 *BP*_*ND*_ values at the peak voxel level in relation to latency to fall (**b**) and print width (**d**). Correlation performed using Spearman’s rank test. Abbreviations: L, left; R, right; *BP*_*ND*_: non-displaceable binding potential; mGluR5: metabotropic glutamate receptor type 5.

VOI-based analysis showed a positive correlation between reduced mGluR5 *BP*_*ND*_ values in the affected striatum and decreased rotarod performance of QA rats at 3 weeks after lesioning (VOI: r = 0.62; *p* = 0.03), which was confirmed by voxel-wise analysis in a cluster in the anterolateral striatum (r = 0.92; *p*_peak_ = 2.02 * 10^−5^ uncorrected, *p*_cluster_ = 0.002; Fig. 4a,b). The compensatory increased print width of the non-affected (ipsilateral) forepaw was correlated to mGluR5 *BP*_*ND*_ values in the contralateral motor cortical region (SPM: r = 0.89; *p*_peak_ = 4.12 * 10^−4^ uncorrected, *p*_cluster_ < 0.001; Fig. 4c,d). No other correlations were observed within VOI- or voxel-based mGluR5 *BP*_*ND*_ values, behavioral tests, and lesion volume.

### Immunohistochemical analysis of the QA-induced striatal lesion

**Figure 5:**
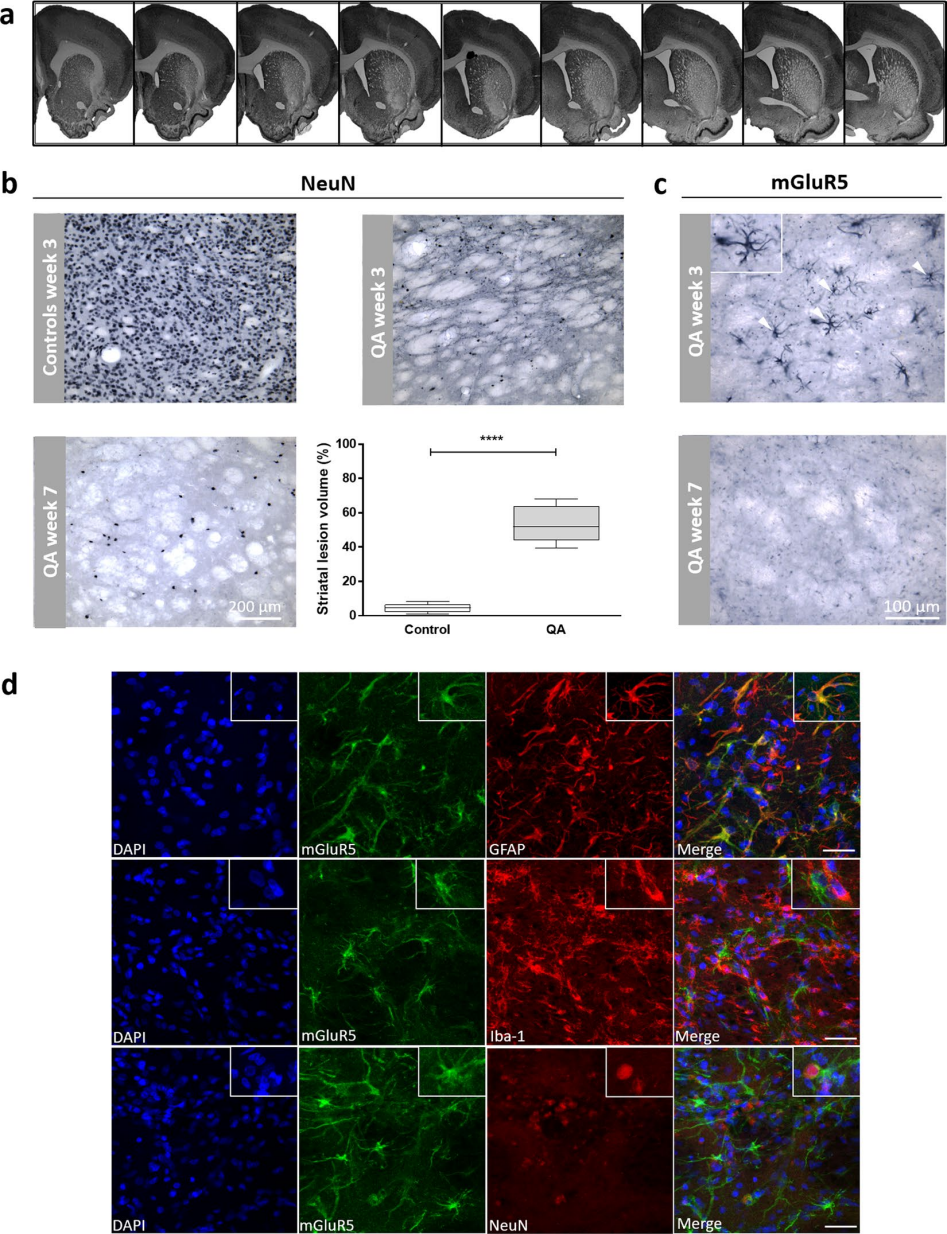
Immunohistochemical characterization of the QA excitotoxic lesion. (**a)** Photomicrographs show a serial representation of coronal sections at ±200 µm interval through the rat striatum, treated with QA at 7 weeks post-injection. Representative NeuN **(b**) and mGluR5 (**C**) staining patterns in coronal sections surrounding the QA injection site in the striatum. The excitotoxic lesion can be visually appreciated by the loss of NeuN immunostaining in the striatum. The striatal lesion volume was determined by delineating NeuN immunoreactivity in controls (n = 5) and QA-lesioned rats (n = 10) and is represented in box plots. ****p < 0.0001; Mann-Whitney U test. Additionally, mGluR5 staining of the lesion core showed mGluR5-positive cells at week 3 (white arrows), which was absent at week 7. **(d)** Representative slices show that the remaining mGluR5 immunostaining largely coincided with GFAP reactivity (inset). Scale bar: 25 µm.

The degeneration of striatal neurons was visualized by the loss of NeuN immunostaining at the site of injection and a quantitative histological analysis was performed to determine the final lesion volume (Fig. 5a,b). QA-lesioned rats depicted an average striatal lesion volume of 52.5 ± 10.7% as compared to 4.3 ± 2.6% in sham-lesioned rats at week 7 (Fig. 5b). In the QA lesion, mGluR5-positive cells were seen in the region of the injection site, but only at week 3 (Fig. 5c) and co-localized predominantly with cells of astrocytic origin (Fig. 5d).

## Discussion

First used by Olney *et al*.^32^, the term excitotoxicity refers to the ability of L-glutamate to induce neuronal dysfunction and, upon chronic exposure, neurodegeneration of post-synaptic structures including both dendrites and cell bodies. Even though this process is largely mediated by ionotropic glutamate receptors, mGluR5 has also been proposed as an important co-factor since chronic activation of the receptor can potentiate NMDA receptor activation and thereby amplify excitotoxicity. Accordingly, targeting of mGluR5 could provide neuroprotection and alleviate cognitive/motor symptoms associated with different neurodegenerative disorders^33^.

In this study, we investigated the link between mGluR5 status, neurodegeneration, and motor symptoms using the pharmacological model employing QA, which is a well-known NMDA receptor agonist with excitotoxic properties. In short, we evaluated mGluR5 receptor alterations in QA-lesioned rats using [^18^F]FPEB longitudinal small-animal PET in combination behavioral assessment and looked at possible correlations between [^18^F]FPEB and disease-associated motor deficits. In addition, we studied QA lesion size and mGluR5 immunoreactivity within different cell types present in the QA lesion.

Compared to sham-injected rats, QA-lesioned rats depicted an *in vivo* decrease in mGluR5 receptor binding in the affected striatum at both time points following stereotactic surgery. This decrease extended towards the ipsilateral globus pallidum and nucleus accumbens at week 7, but not to the contralateral hemisphere or distal anatomical regions. These findings are in line with Ishiwata *et al*., who studied at the adenosine 2A (A2A) receptor, as well as the dopamine D1 and D2 receptors using *in vivo* PET imaging^34^. The A2A receptor is predominantly located at the striatopallidal GABAergic D2 projection neurons, similar to mGluR5^35^. In this study, QA-lesioning led to a reduced uptake of the A2A tracer, comparable to D1 and D2 binding, though the D1 receptor showed a more prominent decline than D2. Previous reports have shown that QA-lesioning targets D1 MSNs of the direct pathway more prominently than D2 MSNs^36^. This could explain why mGluR5 availability remained proportionally higher as compared to the lesion volume, since mGluR5 is predominantly located post-synaptically on the striatopallidal and striatonigral D2 neurons. Striatal QA lesioning has also been utilized as a model for HD since the toxin spares a specific population of striatal neurons that are also spared in HD patients^37^. In accordance with our findings, [^11^C]ABP-688 *BP*_*ND*_ was significantly reduced in the striatum of Q175 transgenic HD mice from 6–13 months old^38^.

Previous research showed a clear astroglial reaction in the affected striatum at 30 days post-injection, with limited extrastriatal astrocytic reaction^39–41^. In line with our histochemical findings in the area of the lesion core, we showed mGluR5-positive cell presence with immune cell-like morphology, which was limited to the 3-week time point. This finding may explain the dynamics of mGluR5 PET since mGluR5 *BP*_*ND*_ continued to decline from week 3 to week 7. It is plausible that astrogliosis, associated with (elevated) mGluR5 expression, could partially mask the decreased mGluR5 signal from neuronal origin. Since mGluR5 is expressed on both microglia and astrocytes, invasion of these cell types during excitotoxicity-induced immune reactions could dampen the reduced mGluR5 contribution from striatal neurons^42–44^. In line with these findings, mGluR5 activation was shown to inhibit microglial NADPH oxidase activity and reduce LPS-induced ROS production, thereby attenuating microglial activation^45,46^. Arguably, we cannot exclude a relative mGluR5 upregulation in the surviving neurons as a compensatory mechanism. In contrast to a previous study from Casteels *et al*., we did not observe any receptor-related alterations in the intact hemisphere, albeit their study studied different targets (using [^18^F]FDG and [^18^F]fallypride) and was performed at different time points and a higher toxin concentration^21^. The authors reported increased glucose metabolism and D2 receptor availability in the contralateral striatum 16–25 weeks post-lesioning. Given that we performed PET imaging at around 2 months following surgery, we might not have been able to detect these adaptive changes in the basal ganglia circuitry, though previous data indicated the degenerative process to be stable at approximately the 2-month time point^40^.

In line with previous findings, QA rats developed significant motor impairment, but no memory deficit. Compared to other studies (>200 nmol toxin), we employed a 120 nmol intrastriatal injection of QA, which leads to partial lesioning and consequently more limited locomotor abnormalities and sensorimotor neglect^37,47,48^. We did notice a worsening of rotarod performance in both treatment groups, which might be due to reduced motivation in the rodents to perform the behavior test^49^. Notwithstanding, we chose to include Sprague-Dawley rats because they are considerably more susceptible to neurological damage and memory impairment as compared to the Wistar Han strain^50^.

Our observations indicated a linear relationship between decreases in mGluR5 receptor binding and reduced rotarod performance at week 3. This relation suggests that higher mGluR5 binding is linked to preserved motor performance, in accordance with our immunohistochemistry findings, which showed that reduced mGluR5 levels coincided with absent NeuN immunostaining. Thus, mGlu5 receptor imaging may reflect the striatal degeneration. However, mGluR5 alterations may also originate from astroglial origin, as suggested by our immunohistochemistry and alternatively, could also be caused by changes in receptor affinity or conformational state. As mentioned before, microglial mGluR5 activation may attenuate activation of the microglia, and potentially limit the extent of the neuronal damage. A limitation to our histological analysis is that vibratome floating sections are not recommended to perform immunofluorescence co-localization experiments because of their slice thickness. However, mGluR5-positive cells in the QA lesion clearly do not have a microglia-like morphology.

We also showed that increased print width of the unaffected forepaw correlated with higher mGluR5 binding in the contralateral motor cortex. VOI and voxel-wise analysis however did not show significant mGluR5 *BP*_*ND*_ differences in this region as compared to saline controls. The role of cortical mGluR5 in locomotor activity was investigated recently and indicates that mGluR5 blockage reduces locomotor activity and rotarod performance^7^. Accordingly, increased print width might be interpreted as a compensatory mechanism namely that the animal supports most of its body weight at the side of the body unaffected by the striatal lesion, possibly leading to an excessive spreading of the digits. Alternatively, the observed correlation may be an (in)direct locomotor effect of the QA-induced striatal lesion. We did not detect a reduced paw intensity or print area of the affected paws.

In conclusion, we found regional changes in mGluR5 availability in the area affected by QA lesioning and limited involvement of extrastriatal regions including the cerebral cortex. We showed a highly significant relation between mGluR5 binding potential and excitotoxicity-mediated neurodegeneration, suggesting that at least part of the mGluR5 alterations were due to the necrotic effect of QA on striatal medium spiny neurons. Altogether, we showed a limited role for mGluR5 in compensatory or plasticity mechanisms to correct post-synaptic distress in the unilateral striatum.

## Data Availability

The datasets generated during and/or analysed during the current study are available from the corresponding author on reasonable request.
